# The ATPase ATP6V1A facilitates rabies virus replication by promoting virion uncoating and interacting with the viral matrix protein

**DOI:** 10.1074/jbc.RA120.014190

**Published:** 2020-11-22

**Authors:** Xing Liu, Fang Li, Jiwen Zhang, Lulu Wang, Jinliang Wang, Zhiyuan Wen, Zilong Wang, Lei Shuai, Xijun Wang, Jinying Ge, Dongming Zhao, Zhigao Bu

**Affiliations:** 1 State Key Laboratory of Veterinary Biotechnology, Harbin Veterinary Research Institute, Chinese Academy of Agricultural Sciences, Harbin, People's Republic of China; 2 National High Containment Laboratory for Animal Diseases Control and Prevention, Harbin, People's Republic of China

**Keywords:** rabies virus, matrix protein, vacuolar ATPase, ATP6V1A, protein–protein interaction, uncoating, RNA interference (RNAi), confocal microscopy, viral replication, ATP6V1A, V-type proton ATPase catalytic subunit A, CCK8, Cell Counting Kit-8, CHX, cycloheximide, CME, clathrin-mediated endocytosis, Co-IP, coimmunoprecipitation, ERA, Evelyn-Rokitnicki-Abelseth, G, glycoprotein, GST, glutathione-*S*-transferase, HDCV, human diploid cell vaccine, L, large polymerase protein, M, matrix protein, MOI, multiplicity of infection, N, nucleoprotein, PCECV, purified chick embryo cell vaccine, PDEV, purified duck embryo vaccine, PVCV, purified Vero cell vaccine, RABV, rabies virus, RNP, ribonucleoprotein, V-ATPase, V-type proton ATPase, VSV, vesicular stomatitis virus

## Abstract

Rabies virus (RABV) matrix protein (M) plays crucial roles in viral transcription, replication, assembly, and budding; however, its function during the early stage of virus replication remains unknown. Here, we mapped the protein interactome between RABV M and human host factors using a proteomic approach, finding a link to the V-type proton ATPase catalytic subunit A (ATP6V1A), which is located in the endosomes where RABV first enters. By downregulating or upregulating ATP6V1A expression in HEK293T cells, we found that ATP6V1A facilitated RABV replication. We further found that ATP6V1A was involved in the dissociation of incoming viral M proteins during viral uncoating. Coimmunoprecipitation demonstrated that M interacted with the full length or middle domain of ATP6V1A, which was dependent on the lysine residue at position 256 and the glutamic acid residue at position 279. RABV growth and uncoating in ATP6V1A-depleted cells was restored by trans-complementation with the full length or interaction domain of ATP6V1A. Moreover, stably overexpressed ATP6V1A enhanced RABV growth in Vero cells, which are used for the production of rabies vaccine. Our findings identify a new partner for RABV M proteins and establish a new role of ATP6V1A by promoting virion uncoating during RABV replication.

Rabies, which is caused by rabies virus (RABV), is an important and devastating infectious disease with an almost 100% mortality rate (1). Annually, about 60,000 people die from rabies and over 15 million people receive post-exposure prophylaxis worldwide (2). RABV belongs to the genus *Lyssavirus* in the Rhabdoviridae family and has a non-segmented negative-strand RNA genome of approximately 12 kb in length. The viral genome consists of five genes, encoding five viral proteins: the nucleoprotein (N), the phosphoprotein (P), the matrix protein (M), the glycoprotein (G), and the large polymerase protein (L) (3, 4). The viral genome is tightly encapsidated by the N protein, which forms the ribonucleoprotein (RNP) and associates with the viral polymerase complex that contains the L and P proteins. The viral capsid is surrounded by host-derived membrane in association with the G and M proteins (5).

The RABV M protein is the smallest of the virion proteins (4) and forms a structural bridge between the virion membrane and the viral RNP (6). The M protein has multiple functions during the mid and late stages of RABV replication. It regulates the balance between viral transcription and replication (7) and functions in viral assembly, budding, and the bullet-shaped morphology of RABV viral particles (8). The M protein exploits different host proteins to achieve its functions. It binds to RelAp43 to inhibit NF-κB–dependent gene expression related to innate immunity in the late stage of virus infection (9, 10). The M protein also mediates the recruitment of host proteins involved in the vacuolar protein sorting pathway to facilitate virus–cell separation (11, 12). The proline-rich motif of the M protein interacts with the WW domains of cellular proteins for viral budding (13). It also targets mitochondria and induces mitochondrial apoptosis during the late stage of virus infection for viral replication and dissemination (14). However, the role of the M protein during the early stage of the RABV life cycle remains unknown.

The first phase of RABV infection is strongly associated with host factors and the biological environment of the infected cells. RABV binds to its receptors and enters host cells via clathrin-mediated endocytosis (CME) (15, 16, 17). Then, trimeric G protein induces membrane fusion of the virion and endosome at low pH (18). Several endosomal ion transports are responsible for the acidification of the endosomal lumen (19, 20). The V-type proton ATPase (V-ATPase) is the sole energizer of endosomal membranes and the major contributor to endosomal acidification, pumping H^+^ from the cytoplasm to the lumen (19, 20). The subunits of the V-ATPase complex form the V0 domain that resides in endosomal membrane and the V1 domain in cytoplasm associated with the V0 domain (21) and play roles in virus replication. RNAi screening revealed that subunits ATP6AP2, ATP6V0B, and ATP6V0D1 of the V-ATPase participate in the infection of several negative-strand RNA viruses, including vesicular stomatitis virus (VSV), lymphocytic choriomeningitis virus, and human parainfluenza virus type 3 (22). Systematic microRNA analysis identified the subunit ATP6V0C as an essential host factor for infectious cytomegalovirus production (23). ATP6V1H binds to the Nef protein of HIV or SIV to internalize CD4 and thus increase viral infectivity (24).

ATP6V1A resides in the V1 domain of the V-ATPase complex and associates with many cellular biological processes in eukaryotic cells (25). ATP6V1A functions as the catalytic subunit of V-ATPase for hydrolyzing ATP to ADP and supplying energy for H^+^ transport (21). ATP6V1A targeted by miR-143 inhibits intracellular salmonella growth in macrophages in pig, demonstrating that ATP6V1A plays important roles in the development of salmonella infection (26). A recent study showed that endogenous cellular microRNA (has-miR-1-3p) used ATP6V1A to inhibit H1N1 replication (27). However, the detailed molecular basis for how ATP6V1A affects viral infection remains unclear. In this study, we identified ATP6V1A as one of the RABV M–interacting host proteins. Our study demonstrated that ATP6V1A facilitates RABV replication by affecting viral uncoating and by interacting with RABV M protein and that RABV M protein plays an important role in the early stage of the RABV life cycle.

## Results

### Mass spectrometry analysis of host proteins coimmunoprecipitated with RABV M protein

**Figure 1:**
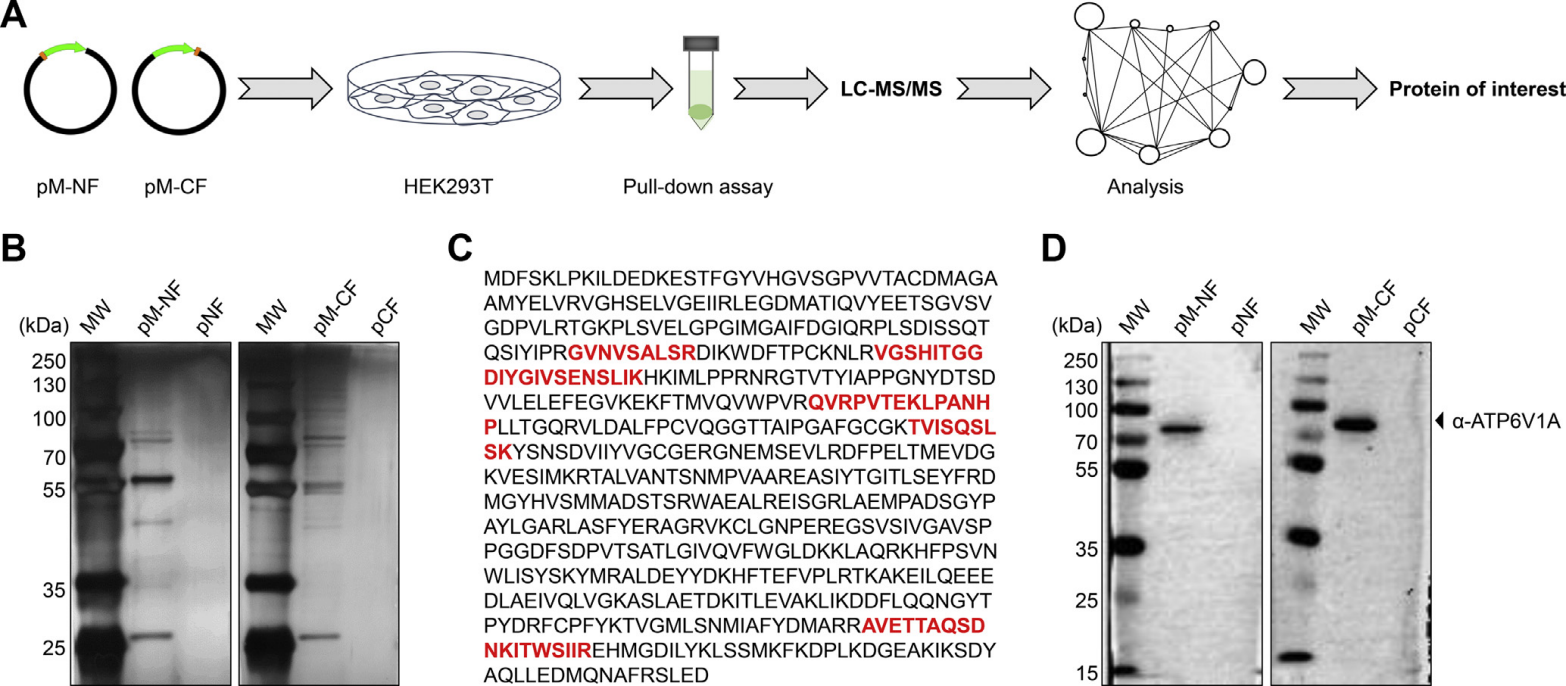
**Proteomic analysis shows that ATP6V1A interacts with RABV M protein.** Pipeline of pull-down assay and LC-MS/MS for screening host proteins that interact with RABV M protein (*A*). HEK293T cells were transfected with pM-NF and pM-CF, which express N- or C-terminally FLAG-tagged M protein, respectively. Affinity-purified host proteins were separated by SDS-PAGE and silver stained (*B*). LC-MS/MS analysis for the identification of ATP6V1A. Peptides matched to the ATP6V1A amino acid sequence are highlighted in *bold red* (*C*). Eluates in (*B*) were separated by SDS-PAGE. ATP6V1A was detected by using an anti-ATP6V1A antibody. MW, protein molecular weight marker in kilodaltons (*D*).

To explore the role of the matrix protein in RABV infection of host cells, we looked for host proteins that interact with the RABV M protein. We performed FLAG tag–based immunoprecipitation of lysates of HEK293T cells transfected with plasmids expressing either N- or C-terminally FLAG-tagged M protein (M-NF or M-CF, respectively) and identified host proteins that interacted with the M proteins by using mass spectrometry (MS) (Fig. 1*A*) as described previously (28). Proteins were detected in lysates of cells transfected with the plasmid expressing FLAG-tagged M proteins but not in those of cells transfected with the control plasmid (Fig. 1*B*). We identified a total of 2672 host proteins that coimmunoprecipitated with the M protein; 492 proteins coimmunoprecipitated with both M-NF and M-CF, 1761 proteins coimmunoprecipitated with M-NF only, and 419 proteins coimmunoprecipitated with M-CF only. The MS data have been deposited in the IMEx consortium through IntAct with the accession code IM-27674 (http://www.imexconsortium.org) (29). The identified host proteins were further analyzed by using the DAVID Gene Functional Classification Tool (https://david.ncifcrf.gov/). The analysis showed that total 38 ATP-powered pump subunits interacted with the M protein (Table S1), which suggests that ATP-powered pumps may play an important role in the life cycle of RABV.

We focused on the catalytic subunit of V-ATPase-ATP6V1A for further study based on previous findings that ATP6V1A influences the pH of endosomes and that the acidic endosomal environment is vital for the early stage of viral infection (18, 21). The amino acid sequence of ATP6V1A is shown in Figure 1*C*, together with five matched peptides detected in the MS analysis (highlighted in red). ATP6V1A was detected in eluted lysates of cells transfected with plasmids expressing FLAG-tagged M proteins but not in those of cells transfected with control vectors, as confirmed by using Western blotting with an ATP6V1A-specific antibody (Fig. 1*D*).

### ATP6V1A is important for RABV replication

**Figure 2:**
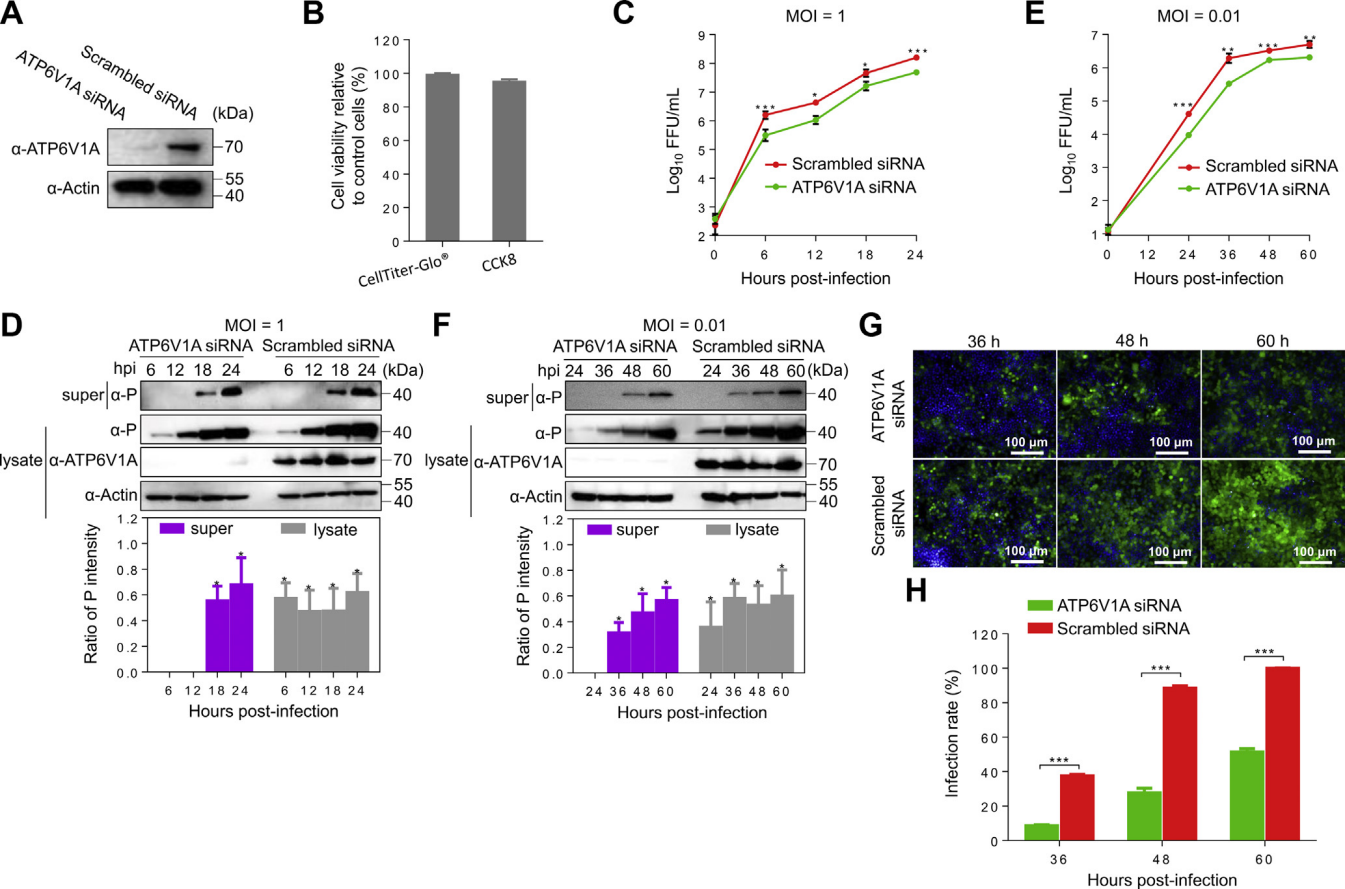
**ATP6V1A knockdown decreases RABV replication.** HEK293T cells were transfected with the indicated siRNAs, and the expression of ATP6V1A was detected by Western blotting (*A*); cell viability following siRNA transfection was determined by the quantification of ATP or NADH using CellTiter-Glo (Promega, USA) reagent or Cell Counting Kit-8 (CCK8) (Dojindo, China), respectively, at 48 h post transfection. The relative assessments of cell viability were normalized by comparison with control cell as 100% cell viability (*B*). HEK293T cells were transfected with the indicated siRNAs and infected with ERA virus at 48 h post transfection. The culture medium of cells was collected and centrifuged (5000*g*/min, 10 min) at 4 °C at indicated time points post infection. Viral titers in the culture supernatants of HEK293T cells infected with ERA virus at an MOI of 1 (*C*) and 0.01 (*E*) were detected after infection. ATP6V1A in the cell lysates and viral P protein in the culture supernatants or lysates of HEK293T cells infected with ERA virus at an MOI of 1 (*D*) and 0.01 (*F*) were detected by Western blotting after infection. Statistical analysis of the P protein levels in the upper panel was performed by using ImageJ software. The value for the P protein was standardized to the β-actin level and normalized to the level of the P protein in cells transfected with scrambled siRNA. HEK293T cells were infected with ERA-eGFP virus at an MOI of 0.01 at 48 h post transfection of siRNA. Cell nuclei were stained with Hoechst 33342 (*blue*). The infectivity of ERA-eGFP virus in cells was examined and observed by using a high content screening system (*G*). Infection ratios were analyzed by using Columbus software (*H*). Data were expressed as means ± SD of triplicate well from one independent experiment. Statistical significance was determined by ANOVA with a multiple comparison correction. ∗*p* < 0.05, ∗∗*p* < 0.01, ∗∗∗*p* < 0.001. lysate, lysate of cells; super, supernatants of culture medium of cells.

To investigate whether ATP6V1A is involved in RABV replication, HEK293T cells were transfected with ATP6V1A siRNA and infected with RABV Evelyn-Rokitnicki-Abelseth (ERA) strain at a multiplicity of infection (MOI) of 1. As a control, additional cells were transfected with a scrambled siRNA and infected with virus (Fig. 2*A*). There was no significant difference in cell viability between ATP6V1A and scrambled siRNA-treated cells based on the quantification of ATP or NADH by using CellTiter-Glo (Promega, USA) reagent or Cell Counting Kit-8 (CCK8) (Dojindo, China), respectively (Fig. 2*B*). Viral titration with cell supernatants showed that ATP6V1A knockdown decreased viral titers by 5.1-fold at 6 h post infection and decreased RABV replication in HEK293T cells at all later times post infection (Fig. 2*C*). The RABV P protein in cell supernatants and lysates was detected by Western blotting at different times post infection. Viral P protein in cell supernatants was first detected at 18 h post infection, with a considerable reduction in ATP6V1A-knocked-down cells compared with that in control cells (Fig. 2*D*). ATP6V1A knockdown resulted in less accumulation of viral proteins in cells (Fig. 2*D*). We also infected ATP6V1A-knocked-down and scrambled siRNA-transfected HEK293T cells with ERA virus at an MOI of 0.01. The results showed that knockdown of ATP6V1A decreased RABV replication in the supernatant of infected cells by 6-fold at 36 h post infection (Fig. 2*E*). Strikingly, the P protein was almost undetectable at 36 h post infection in the culture supernatants of ATP6V1A-knocked-down cells but was clearly detectable in the culture supernatants of the control cells (Fig. 2*F*), indicating that the knockdown of ATP6V1A suppressed viral replication.

To further confirm the role of ATP6V1A in RABV replication, we infected ATP6V1A-knocked-down and scrambled siRNA-transfected HEK293T cells with ERA-eGFP, a recombinant RABV ERA strain that expresses enhanced green florescence protein, at an MOI of 0.01. Cells expressing eGFP were seen as indicators of viral infection by using a high-content imaging-based screen as described previously (30). The results showed that the infectivity of ERA-eGFP dramatically decreased in ATP6V1A-knocked-down cells compared with that in control cells post infection (Fig. 2*G*) by 29%, 61%, and 49% at 36, 48, and 60 h post infection, respectively (Fig. 2*H*).

**Figure 3:**
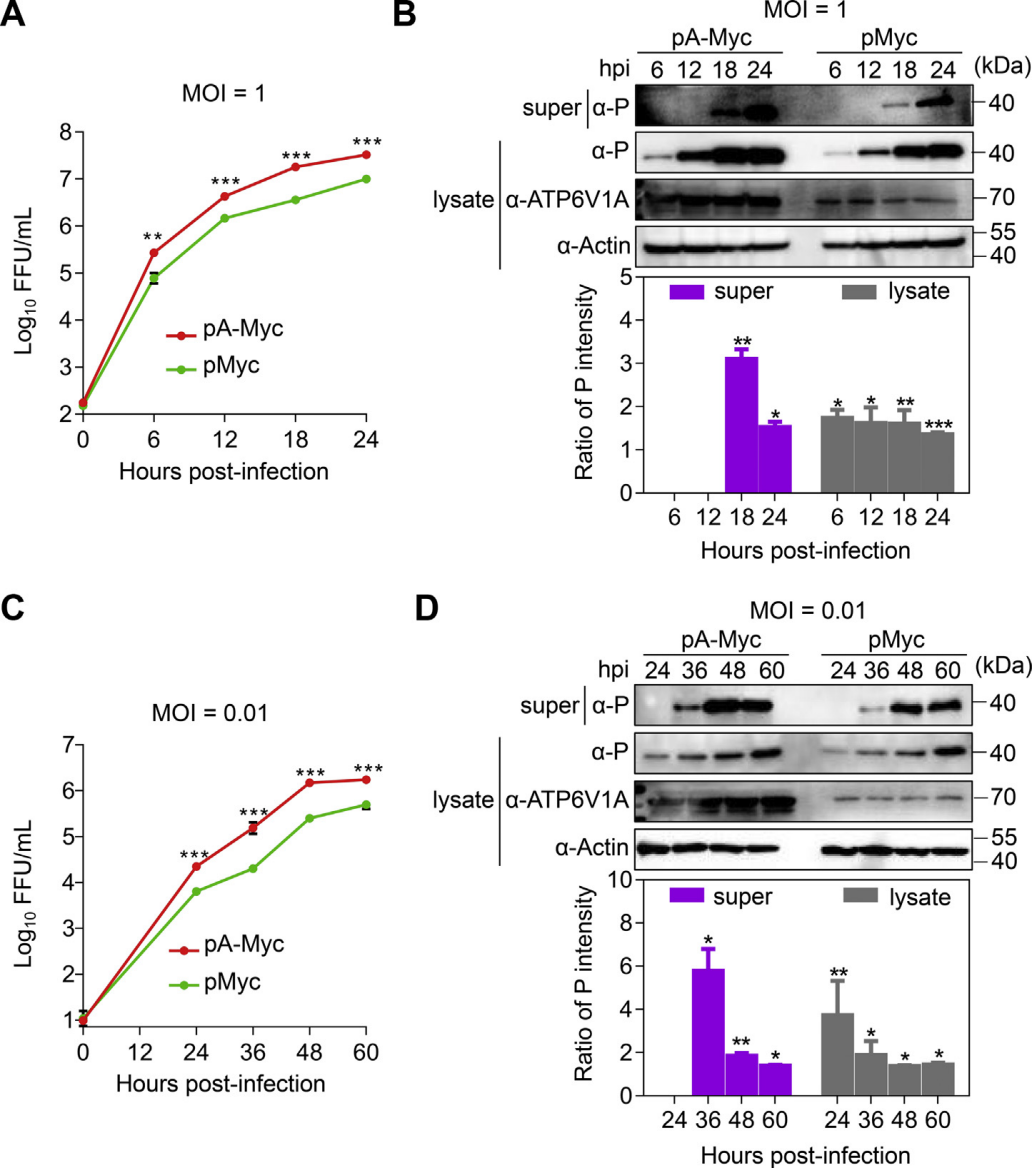
**Overexpression of ATP6V1A enhances RABV replication.** HEK293T cells were transfected with pA-Myc and infected with ERA virus at an MOI of 1 and 0.01 at 24 h post transfection. The culture medium of cells was collected and centrifuged (5000*g*/min, 10 min) at 4 °C at indicated time points post infection. Viral titers in the culture supernatants of HEK293T cells infected with ERA virus at an MOI of 1 (*A*) and 0.01 (*C*) were detected after infection. ATP6V1A in the cell lysates and viral P protein in the culture supernatants or lysates of HEK293T cells infected with ERA virus at an MOI of 1 (*B*) and 0.01 (*D*) were detected by Western blotting after infection. Statistical analysis of the level of P protein in the upper panel was performed by using ImageJ software. The value for the P protein was standardized to the β-actin level and normalized to the level of the P protein in cells transfected with the control plasmid pMyc. Data were expressed as means ± SD of triplicate well from one independent experiment. Statistical significance was determined by ANOVA with a multiple comparison correction. ∗*p* < 0.05, ∗∗*p* < 0.01, ∗∗∗*p* < 0.001. lysate, lysate of cells; super, supernatants of culture medium of cells.

We then evaluated whether overexpression of ATP6V1A affects RABV replication. HEK293T cells transfected with pA-Myc to overexpress ATP6V1A were infected with ERA virus at an MOI of 1 at 24 h post transfection. Virus and P protein in the cell supernatants and lysates were titrated or detected at different times. The results showed that overexpression of ATP6V1A promoted the growth of ERA virus and increased the viral titers by 5-fold at 18 h post infection (Fig. 3*A*). The expression of P protein was primarily detected at 6 h post infection in the cell lysates and at 18 h post infection in the cell supernatants, and the expression level increased substantially over time in the cells overexpressing ATP6V1A (Fig. 3*B*). Overexpression of ATP6V1A also enhanced RABV growth in cells infected with ERA virus at an MOI of 0.01. Viral titers in the supernatants of cells overexpressing ATP6V1A were roughly 1-log higher than those of control cells at 36 and 48 h post infection (Fig. 3*C*). Viral P protein expression in cell supernatants or lysates was also enhanced by 7-fold in cells overexpressing ATP6V1A at 36 h post infection, which was consistent with the observed enhanced virus growth (Fig. 3*D*). These results suggest that ATP6V1A is important for the replication of RABV in cells.

### ATP6V1A is critical for efficient RABV uncoating

**Figure 4:**
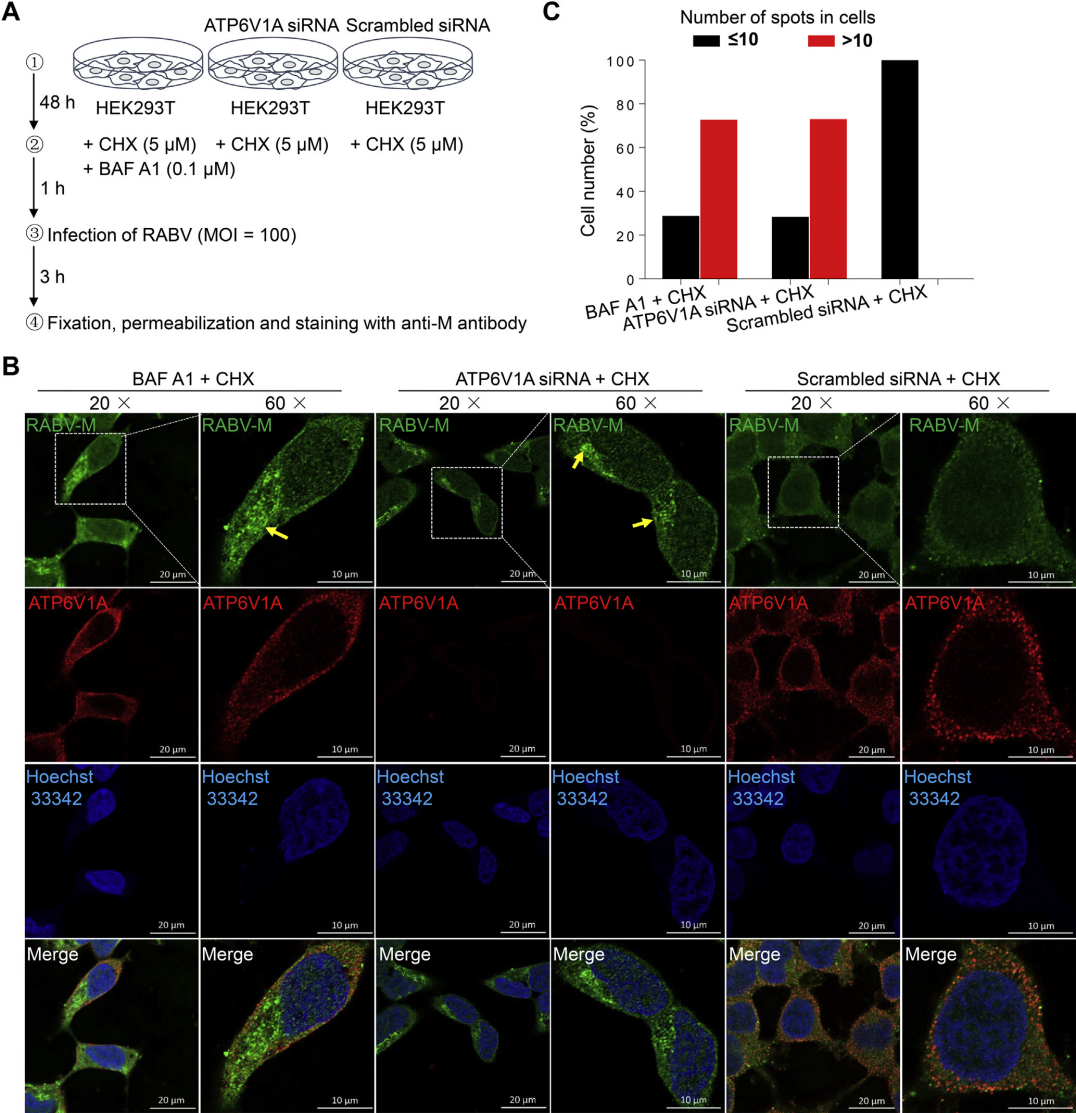
**ATP6V1A knockdown affects the dissociation of M proteins.** Schematic of detection of incoming M proteins during the early stage of RABV life cycle (*A*). HEK293T cells were transfected with the indicated siRNAs or treated with BAF A1 as a control. At 48 h post transfection, cells were infected with ERA virus at an MOI of 100 in the presence of CHX. Three hours later, RABV M protein (*green*), endogenous ATP6V1A (*red*), and cell nuclei (*blue*) were stained and observed for aggregates of RABV M proteins. *Yellow solid arrows* indicate high-intensity punctate staining of M proteins (*B*). Quantitative analysis of the dissociation of incoming RABV M proteins in virus-infected cells. On the basis of the confocal microscopy in *B*, the infected cells at 3 h post infection was categorized into two types, containing more than 10 high-intensity punctate staining spots in each cell, or containing less than 10 spots in each cell. The results shown were calculated from 130 cells under a confocal microscope with a 20× objective lens (*C*).

RABV enters cells through receptor-mediated CME. Virus-endosome membrane fusion is stimulated by RABV G proteins, which are conformationally changed after being exposed to acidic conditions in the endosome (18). After membrane fusion, incoming viral M proteins dissociate and release viral genome to cytoplasm (31). To measure the effect of ATP6V1A on the early stage of the RABV life cycle, HEK293T cells were transfected with ATP6V1A-specific siRNA to knockdown endogenous ATP6V1A and were then infected with RABV ERA in the presence of the protein synthesis inhibitor cycloheximide (CHX) to prevent the synthesis of new viral proteins. Successful membrane fusion and uncoating in endosome results in the release of the virion contents into the cytoplasm, which can be evaluated by monitoring the diffuse distribution of the incoming viral M proteins by immunofluorescence. BAF A1 was used as a control to hinder the internalization of RABV into the cytoplasm. As an inhibitor of the H^+^-ATPase pump, BAF A1 blocks endosomal acidification and eliminates the trigger of the conformational rearrangements in the RABV G protein that are necessary for membrane fusion (18, 32) (Fig. 4*A*). ATP6V1A distributed in the cytoplasm, which was consistent with previous report (25). In the presence of CHX, high-intensity punctate staining was observed in the ATP6V1A-knocked-down cells and BAF A1-treated cells but not in the cells transfected with scrambled siRNA (Fig. 4*B*), indicating that more virus particles remained intact in the ATP6V1A-knocked-down cells. To quantify the dissociation of RABV M proteins in virus-infected cells, we chose 130 cells from each group to count high-intensity punctate staining spots by using Imaris software (Bitplane AG, Switzerland) and found that more than 70% of the cells treated with BAF A1, or transfected with ATP6V1A siRNA, contained more than 10 high-intensity punctate staining spots in each cell (Fig. 4*C*) and maintained RABV particles without uncoating. No cells transfected with scrambled siRNA contained more than 10 high-intensity punctate staining spots (Fig. 4*C*). Taken together, these data demonstrate that ATP6V1A knockdown inhibits the dissociation of RABV M proteins during the early stage of viral life cycle.

**Figure 5:**
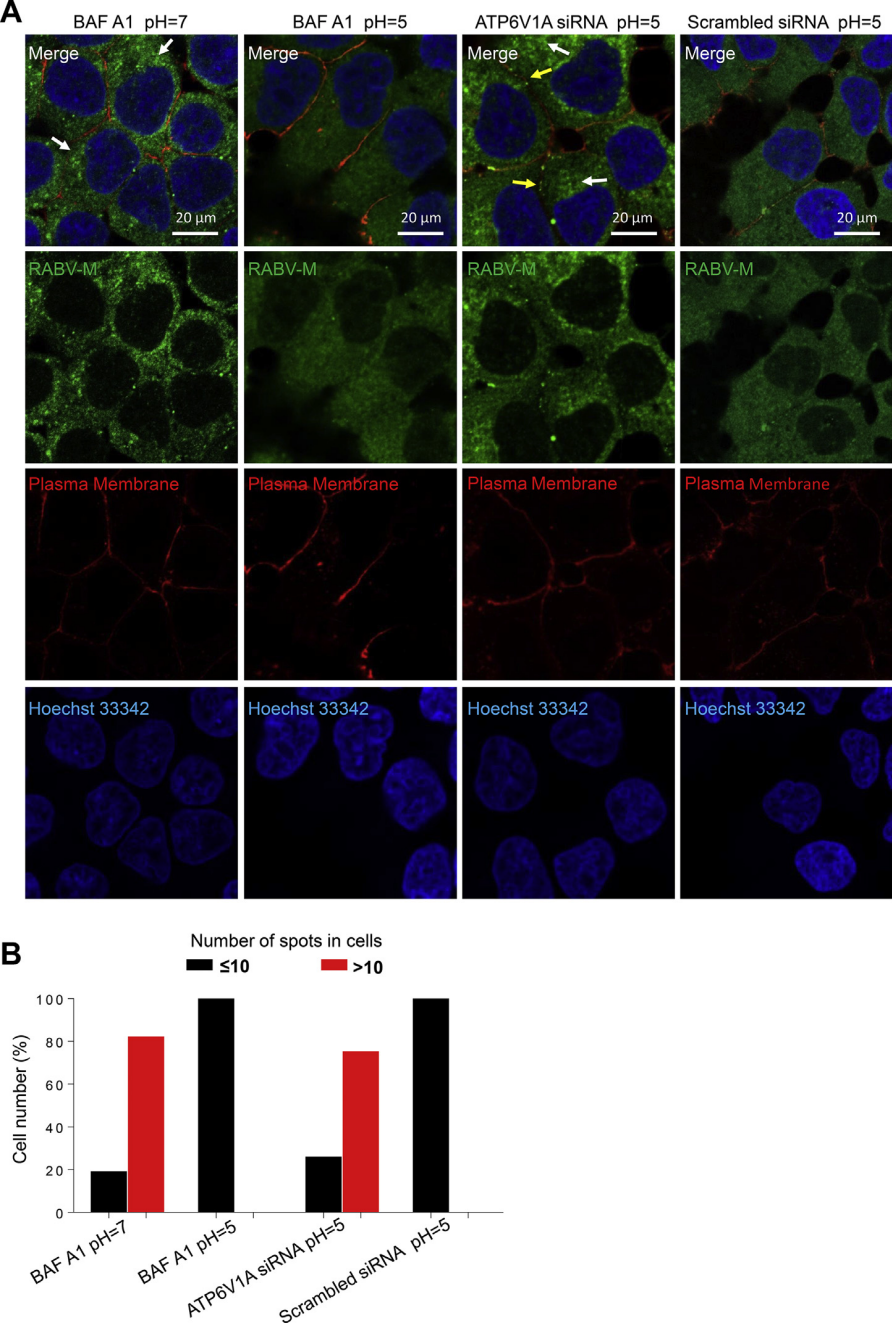
**ATP6V1A is required for RABV uncoating.** HEK293T cells were transfected with the indicated siRNA for 48 h, treated with BAF A1 or not for 1 h, and later infected with ERA for 1 h on ice in the presence of CHX. Cells were then treated with PBS (pH 5 or 7.2) for 15 min, washed, and incubated at 37 °C for 2 h in the presence of CHX. Next, 12.5 μg/ml Alexa Fluor 594-conjugated Wheat Germ Agglutinin (Thermo Invitrogen, USA) was added at 37 °C for 10 min before fixation to stain plasma membrane. RABV M protein (*green*), cell plasma membrane (*red*), and cell nuclei (*blue*) were stained and observed for aggregation of M proteins by confocal microscopy. White or yellow solid arrows indicate the high-intensity punctate staining of aggregated M proteins located on cytoplasm or cell plasma membrane, respectively (*A*). Quantitative analysis of RABV uncoating in virus-infected cells. On the basis of the confocal microscopy in *A*, the infected cells at 3 h post infection were categorized into two types, containing more than 10 high-intensity punctate staining spots in each cell, or containing less than 10 spots in each cell. The results shown were calculated from 130 cells under a confocal microscope with a 20× objective lens (*B*).

Viral M proteins dissociate and release from vRNPs in the step of RABV uncoating (33). RABV, similar to many viruses including West Nile virus, Sindbis virus, and influenza A virus, can enter cells to trigger the fusion of viral envelope and cell membrane without endosome acidification regulated by V-ATPase if the environment is acidic (pH = 5) (18, 34, 35, 36, 37). This is termed an acid bypass assay, allowing us to detect the effect of ATP6V1A on the step of RABV uncoating after membrane fusion as previously described (38). Viral M proteins were high-intensity punctate staining in cells treated with BAF A1 and incubated with neutral PBS; whereas when treated with acidic PBS to allow membrane fusion at the low pH, the high-intensity immunofluorescence staining of viral M proteins disappeared, indicating that the virus already uncoated (Fig. 5*A*). Under acidic conditions allowing membrane fusion, depletion of ATP6V1A still inhibited the dissociation of viral M proteins in particles, indicating that ATP6V1A plays a role in RABV uncoating after membrane fusion (Fig. 5*A*); the observation also found that, although a small amount of aggregated M proteins remained in cell plasma membrane, most of the aggregated M proteins trafficked to the cytoplasm (Fig. 5*A*). The dissociation of RABV M proteins was quantified by using Imaris software as described above, and the results showed that 82% of the cells treated with BAF A1 in neutral conditions contained more than 10 spots in each cell (Fig. 5*B*), whereas no cells treated with BAF A1 in acidic conditions had more than 10 spots (Fig. 5*B*), which indicated that virion under acidic conditions bypassed endocytic requirements and completed membrane fusion directly. However, 75% of the cells transfected with ATP6V1A siRNA and incubated in acidic conditions still contained more than 10 spots in each cell (Fig. 5*B*), whereas no cells transfected with scrambled siRNA contained more than 10 spots (Fig. 5*B*). Collectively, these findings suggest that ATP6V1A is required for RABV uncoating.

### ATP6V1A and M protein interact directly

**Figure 6:**
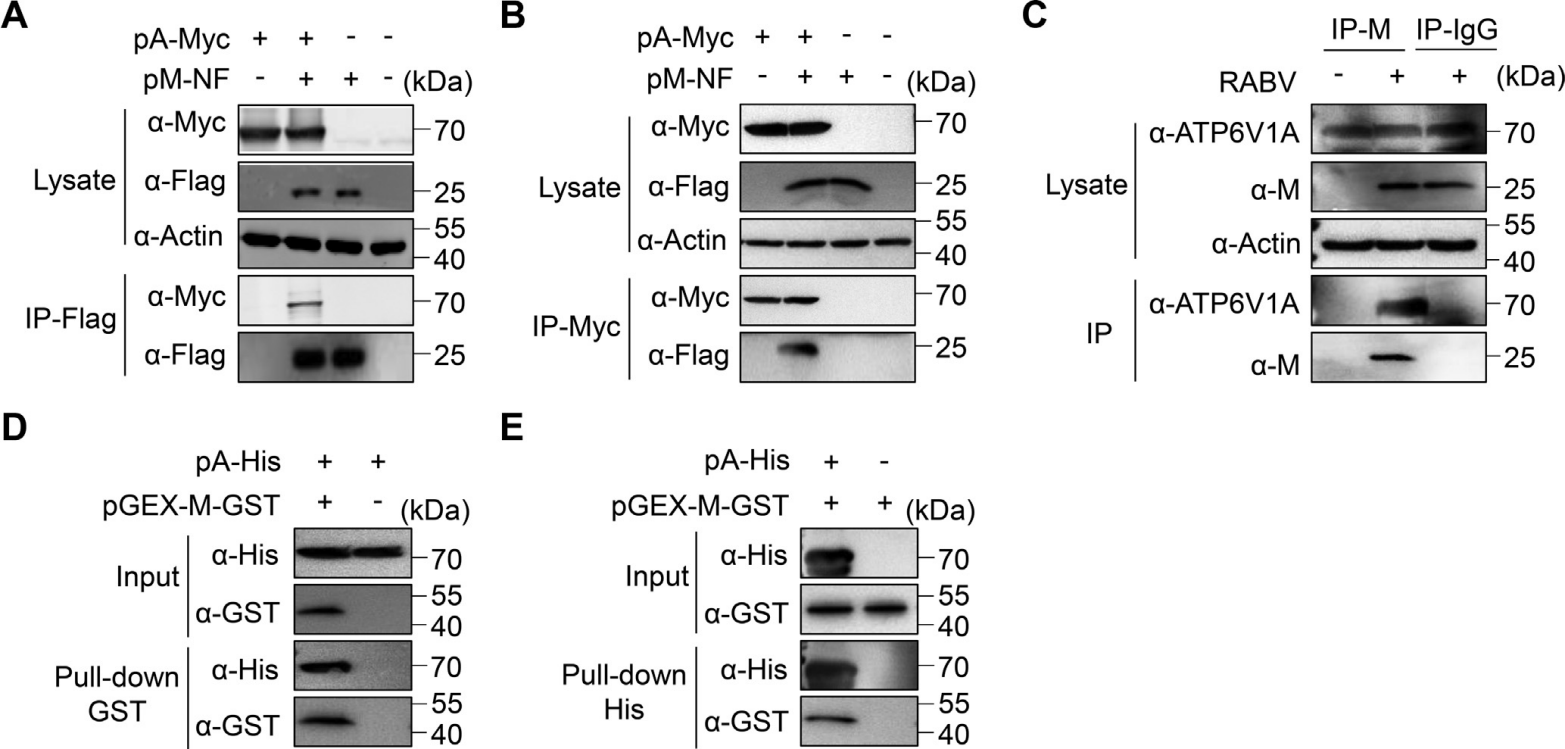
**Interaction of ATP6V1A with RABV M protein.** HEK293T cells were cotransfected with pA-Myc and pM-NF. At 24 h post transfection, cell lysates were collected for coimmunoprecipitation with beads conjugated with FLAG antibody (*A*) and Myc antibody (*B*). HEK293T cells were infected with ERA virus at an MOI of 5 or not, and the interaction of endogenous ATP6V1A and M protein in the cell lysates was detected by coimmunoprecipitation with antibody against M proteins rather than IgG and protein A-conjugated beads (*C*). Purified M tagged with GST (M-GST) from *E. coli* and ATP6V1A tagged with His (ATP6V1A-His) from *E. coli* were used as input fractions to perform the GST pull-down for ATP6V1A-His (*D*) and the His pull-down for M-GST (*E*) in the lower panel.

To further examine the interaction between ATP6V1A and the M protein, we performed coimmunoprecipitation (Co-IP) and Western blotting analysis. HEK293T cells were transfected with plasmids expressing FLAG-tagged RABV M (pM-NF) and Myc-tagged ATP6V1A (pA-Myc) together, or individually. Myc-tagged ATP6V1A was eluted and coimmunoprecipitated with FLAG-tagged M protein but not FLAG peptide when they were coexpressed (Fig. 6*A*). In addition, FLAG-tagged M protein was coimmunoprecipitated with Myc-tagged ATP6V1A in a reverse Co-IP experiment (Fig. 6*B*). These results indicate that ATP6V1A interacts with RABV M protein in cells.

Next, we asked whether endogenous ATP6V1A interacts with RABV M protein during RABV infection. HEK293T cells were infected with ERA virus at an MOI of 5 or uninfected. At 24 h post infection, we performed the Co-IP assay with lysates of infected cells and beads conjugated with IgG or antibodies to M protein. Endogenous ATP6V1A was immunoprecipitated with RABV M protein in virus-infected cell lysates (Fig. 6*C*). These results suggest that RABV M protein interacts with endogenous ATP6V1A during RABV replication.

To determine whether the interaction between M and ATP6V1A was physically direct, we expressed and purified C-terminal glutathione-*S*-transferase (GST)-fused M protein and C-terminal His-tagged ATP6V1A. We then used GST pull-down and immunoprecipitation analysis to reveal that GST-fused M protein directly binds to His-tagged ATP6V1A (Fig. 6*D*). This direct interaction was confirmed with His-tagged pull-down that GST-fused M protein immunoprecipitated with His-tagged ATP6V1A (Fig. 6*E*). Our findings prove that ATP6V1A directly interacts with M protein.

### Amino Acids 160 to 309 of ATP6V1A Are Responsible for the Interaction with M Protein

**Figure 7:**
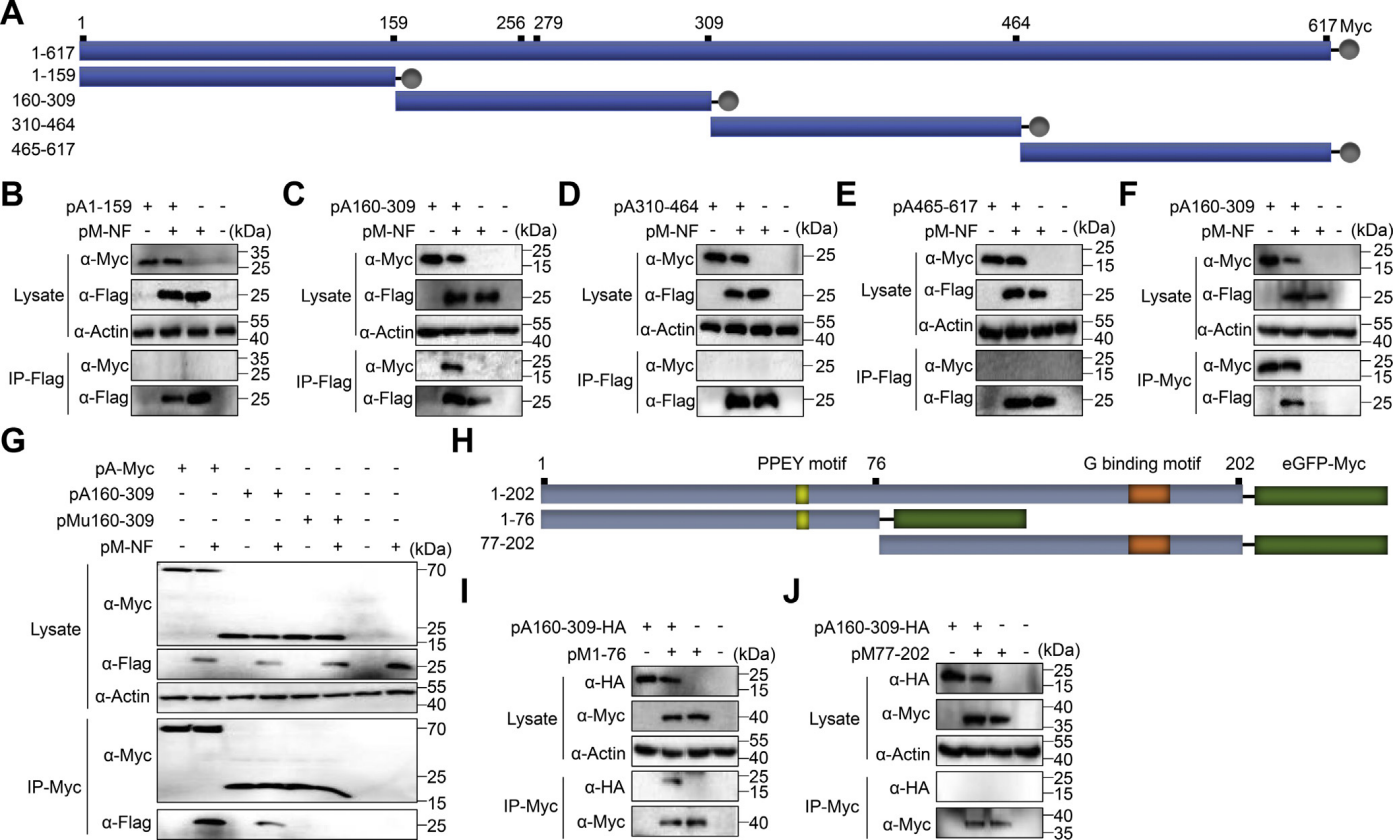
**Identification of the domains of ATP6V1A and the M protein involved in their interaction.** The fragment of amino acids 160 to 309 of ATP6V1A interacts with M protein. Schematic of the ATP6V1A truncates tagged with Myc and locations of the amino acid residues are noted (*A*). HEK293T cells were cotransfected with pM-NF and truncated constructs of ATP6V1A (i.e., pA1-159, pA160–309, pA310–464, or pA465–617), and the cell lysates were collected for coimmunoprecipitation assays using beads conjugated with anti-FLAG antibody (*B–E*) for FLAG-tagged M or Myc antibody for Myc-tagged ATP6V1A or its truncates (*F* and *G*). The N-terminal domain of the M protein interacts with ATP6V1A. Schematic of the M protein and its truncates fused to eGFP tagged with Myc, and the locations of the amino acids are noted (*H*). HEK293T cells were cotransfected with pA160-309-HA and the truncated M constructs pM1-76 (*I*) and pM77-202 (*J*); cell lysates were collected for coimmunoprecipitation assays using beads conjugated with Myc antibody.

To define the key domain in ATP6V1A that determines its interaction with M protein, we generated four truncated ATP6V1A constructs, pA1-159, pA160-309, pA310-464 and pA465-617, that, respectively, expressed amino acids 1 to 159, 160 to 309, 310 to 464, and 465 to 617, and fused each of them with a Myc-tag on the C terminus (Fig. 7*A*). The four constructs were, respectively, cotransfected with pM-NF into HEK293T cells. At 24 h post transfection, cell lysates were prepared for Co-IP assays with beads conjugated with mAbs to FLAG. All four truncated constructs of ATP6V1A were well expressed (Fig. 7, *B*–*E*), and only the fragment of amino acids 160 to 309 of ATP6V1A (A160-309) (Fig. 7*C*) interacted with the M protein. We also performed the reverse Co-IP assay using beads conjugated with mAbs to Myc and confirmed that also only A160-309 coimmunoprecipitated with the M protein (Fig. 7*F*). These results demonstrate that the middle domain of ATP6V1A, namely, amino acids 160 to 309, is responsible for the interaction of ATP6V1A with the M protein.

### Mutations (K256Q/E279Q) in A160-309 abolish its interaction with the M protein

Previous studies have shown that the K256Q mutation in ATP6V1A results in an unstable V-ATPase complex and that the E279Q mutation affects primary ATP hydrolysis (39). Interestingly, these two key amino acids are both located in A160-309, which interacts with the RABV M protein. We confirmed that the two single mutants are not enough to destroy the interaction of A160-309 with M protein (data not shown). To examine whether the double mutations affect the interaction between A160-309 and the M protein, we generated the construction pMu160-309 to express a mutant of A160–309 with K256Q/E279Q (Mu160–309). HEK293T cells were cotransfected with pMu160-309 and pM-NF and lysed at 24 h post transfection for the Co-IP assay. The results showed that Mu160-309 lost the ability to interact with the M protein (Fig. 7*G*), suggesting that the residues at positions 256 and 279 in A160-309 are critical for the interaction with the M protein.

### The N-terminal domain of the M protein interacts with truncated A160-309

To define the domain of the M protein that interacts with A160-309, we generated two constructs, pM1-76 and pM77-202, that, respectively, expressed two truncated M protein fragments, namely, amino acids 1 to 75 (M1-76) and amino acids 77 to 202 (M77–202) (Fig. 7*H*). Both truncates were fused to the C-terminal eGFP that was tagged with Myc. We, respectively, transfected pM1-76 and pM77-202 with pA160-309-HA into HEK293T cells and lysed them at 24 h post transfection for the Co-IP assay. The results showed that the N-terminal domain of the M protein interacts with A160-309 (Fig. 7*I*) rather than the C-terminal domain (Fig. 7*J*).

### *Trans*-complementation of ATP6V1A or A160-309 recovers its promotion to RABV replication in ATP6V1A knockdown cells

**Figure 8:**
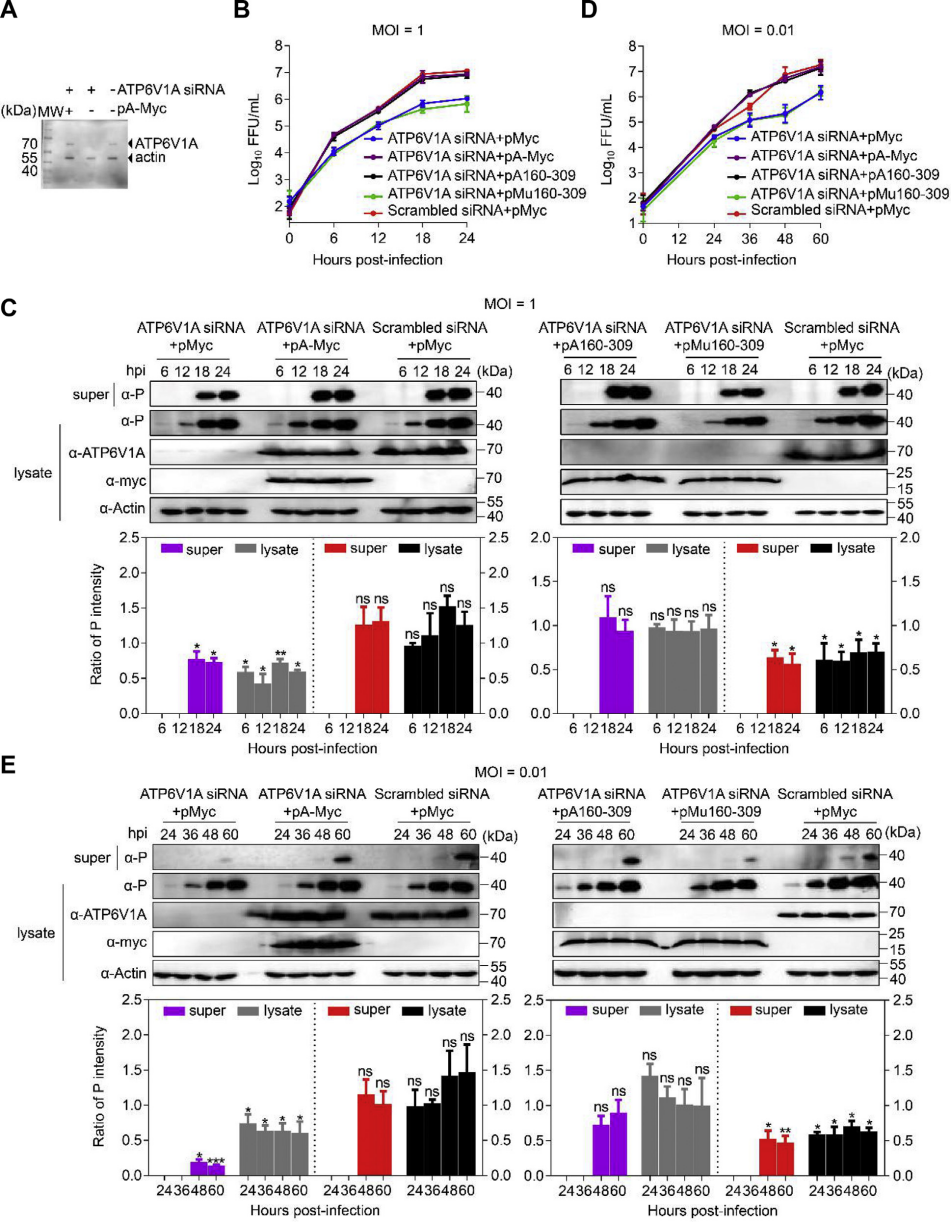
**Transfection of ATP6V1A or its truncate *trans*-complements the decrease in ERA replication in cells transfected with RNAi of ATP6V1A.** HEK293T cells were transfected with ATP6V1A or scrambled siRNA. At 48 h post transfection of siRNAs, cells were transfected with pA-Myc, and the ATP6V1A protein level was detected by Western blotting at 24 h post transfection (*A*). At 48 h post transfection of siRNAs, cells were transfected with pA-Myc, and pA163-309, or pMu160-309, and infected with ERA virus at an MOI of 1 (*B* and *C*) or 0.01 (*D* and *E*) at 24 h post transfection. The culture medium of cells was collected and centrifuged (5000*g*/min, 10 min) at 4 °C at indicated time points post infection. Viral titers in the culture supernatants of the cells were detected at different times after infection (*B* and *D*). ATP6V1A and its truncates in the cell lysates and viral P protein in the culture supernatants and lysates of the HEK293T cells were detected by Western blotting. The ratio of viral P protein in the culture supernatant (*purple bar*) or cellular lysate (*gray bar*) of cells transfected with ATP6V1A siRNA and pMyc (left panel) or pA160-309 (right panel) was compared with that of cells transfected with Scrambled siRNA and pMyc. The ratio of viral P protein in the culture supernatant (*red bar*) or cellular lysate (*black bar*) of cells transfected with ATP6V1A siRNA and pA-Myc (left panel) or pMu160-309 (right panel) was compared with that of cells transfected with Scrambled siRNA and pMyc (*C* and *E*). Statistical analysis of the P protein levels in the upper panel was performed by using ImageJ software. The value for the P protein in the culture supernatants or lysates of the cells was standardized to the β-actin level and normalized to the level of the P protein in the culture supernatants or lysates of the cells transfected with the scrambled siRNA and control vector pMyc, respectively. Data were expressed as means ± SD of triplicate well from one independent experiment. Statistical significance was determined by ANOVA with a multiple comparison correction. ∗*p* < 0.05, ∗∗*p* < 0.01, ∗∗∗*p* < 0.001. lysate, lysate of cells; super, supernatants of culture medium of cells.

To verify the effect of ATP6V1A on the replication of RABV, HEK293T cells were transfected with siRNA to suppress the expression of endogenous ATP6V1A. At 48 h post transfection of siRNA, cells were transfected with pA-Myc, pA160-309, pMu160-309, or pMyc. At 24 h post transfection, cells were infected with ERA virus at an MOI of 1 or 0.01. Western blotting showed that transfection with pA-Myc recovered ATP6V1A expression in siRNA-transfected cells (Fig. 8*A*). Transfection with pA-Myc or pA160-309 obviously enhanced virus replication in ATP6V1A RNAi cells compared with pMu160-309 or control vector pMyc at an MOI of 1 or 0.01 (Fig. 8, *B–E*). There was no significant difference in ERA virus titers between the *trans-*complementation of pA-Myc and pA160-309 or pMu160-309 and control vector pMyc.

Transfection of pA-Myc and pA160-309 also resulted in more than 8-fold higher expression of the P protein compared with transfection of pMu160-309 or the control vector pMyc in ATP6V1A RNAi cells that were infected with ERA virus at an MOI of 0.01 at 60 h post infection (Fig. 8, *D* and *E*), which is comparable with that in mock knockdown cells. The results demonstrate that *trans*-complementation with ATP6V1A or A160-309 restores the effect of ATP6V1A on RABV replication in ATP6V1A-knocked-down cells.

### *Trans*-complementation of ATP6V1A or A160-309 recovers RABV uncoating

**Figure 9:**
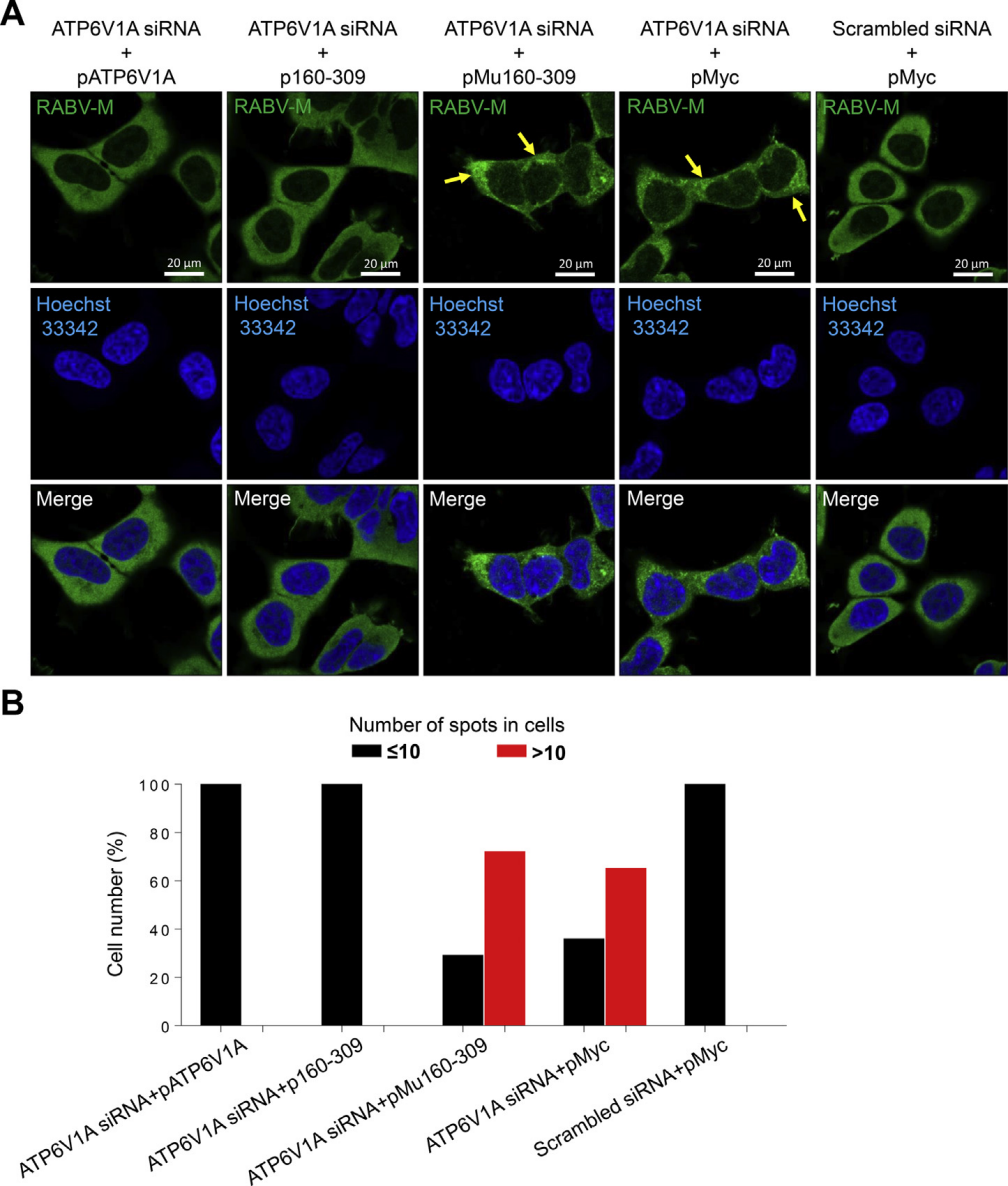
**Transfection of ATP6V1A or its truncate *trans*-complements decreased ERA uncoating in ATP6V1A depleted cells.** HEK293T cells were transfected with ATP6V1A siRNA or scrambled siRNA. At 48 h post transfection of siRNA, cells were transfected with the indicated plasmids for 24 h and infected with ERA at an MOI of 100 for 1 h on ice. Cells were then treated with PBS (pH 5) for 15 min, washed, and incubated at 37 °C for 2 h in the presence of cycloheximide. RABV M protein (*green*) and cell nuclei (*blue*) were stained and observed for M protein aggregation by confocal microscopy. *Yellow solid arrows* indicate high-intensity punctate staining of M proteins (*A*). Quantitative analysis of RABV uncoating in virus-infected cells. On the basis of the confocal microscopy in *A*, the infected cells at 3 h post infection was categorized into two types, containing more than 10 high-intensity punctate staining spots in each cell, or containing less than 10 spots in each cell. The results shown were calculated from 130 cells under a confocal microscope with a 20× objective lens (*B*).

To test the effect of ATP6V1A on RABV uncoating, we performed *trans*-complementation assay as described above. The cells were infected with ERA at an MOI of 100 and treated with acidic PBS for acid bypass assay as previously described (38). We found that, in the cells *trans*-complemented with Mu160-309, the viral M protein was aggregated together with high-intensity punctate staining, which was also observed in the cells transfected with pMyc (Fig. 9*A*), whereas in the cells *trans*-complemented with ATP6V1A or A160-309, the high-intensity punctate staining of viral M protein disappeared, indicating that the virion was uncoated (Fig. 9*A*). In acidic condition allowing membrane fusion of RABV, *trans*-complementation of ATP6V1A mutant failing to interact with M protein was unable to recover the dissociation of viral M protein during uncoating after membrane fusion (Fig. 9*A*). To quantify the dissociation of RABV M proteins, we used Imaris software to count high-intensity punctate staining spots in 130 virus-infected cells from each group. The results showed that, in acidic conditions, more than 65% of the cells *trans*-complemented Mu160-309 or without *trans*-complementation still contained more than 10 high-intensity punctate staining spots (Fig. 9*B*), whereas no cell has more than 10 spots when it *trans*-complemented with ATP6V1A or A160-309 (Fig. 9*B*). These findings further indicated that ATP6V1A participates in the replication and uncoating of RABV, which is associated with its interaction with M protein.

### ATP6V1A knockdown has no effect on the production of rabies VLPs

**Figure 10:**
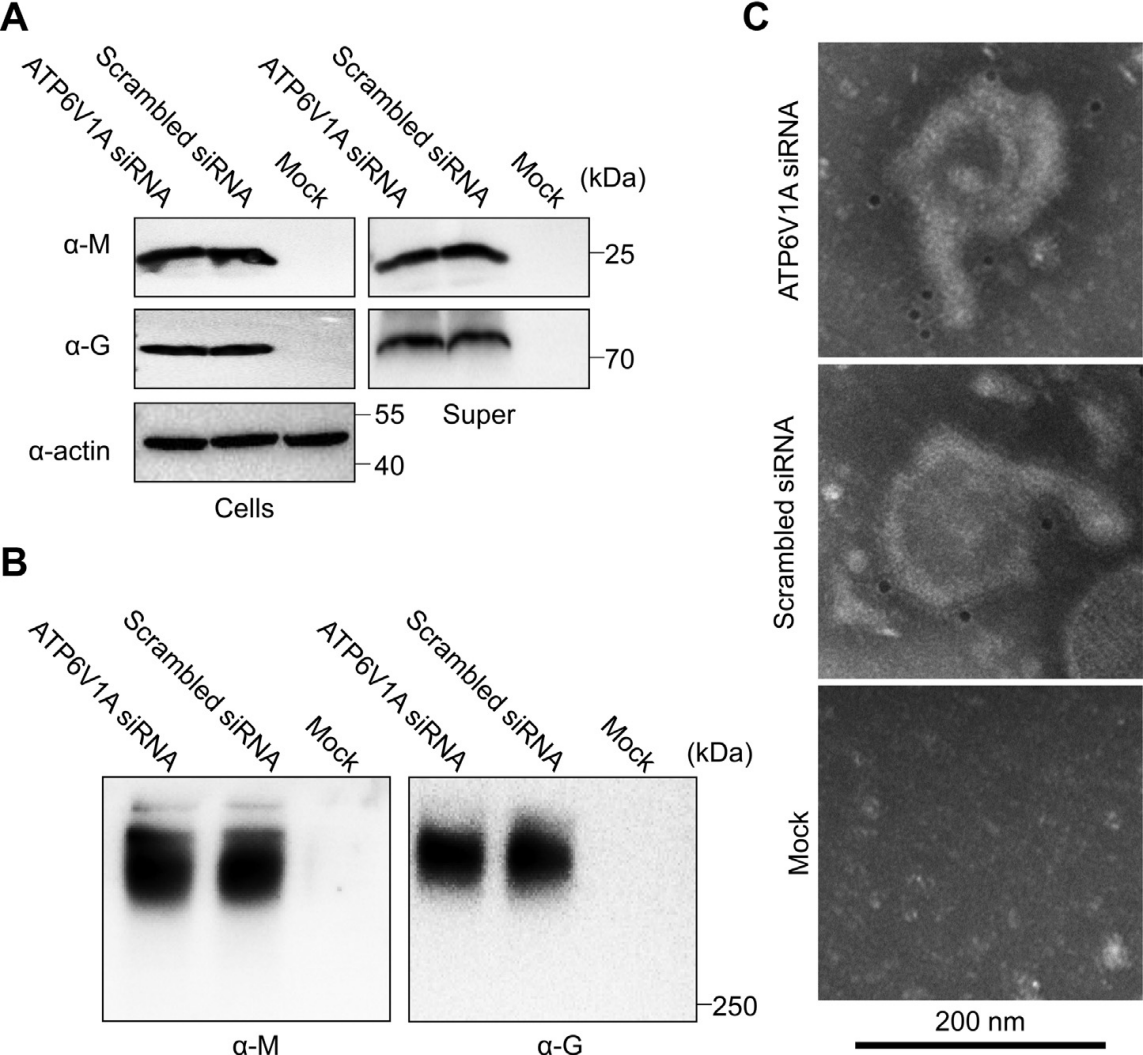
ATP6V1A knockdown has no effect on the production of rabies VLPs. HEK293T cells were transfected with ATP6V1A siRNA or scrambled siRNA for 48 h, and then transfected with plasmids of pcM and pcG at molar ratio of 6:1. At 48 h post transfection, cell culture supernatants were harvested and ultracentrifuged. Purified rabies VLPs were detected by Western blotting with murine serum against RABV M or G protein in denaturing conditions (*A*) or non-denaturing and non-reducing conditions (*B*). Purified rabies VLPs were incubated with mouse anti-G serum and gold-labeled goat anti-mouse IgG antibody, then stained with 2% phosphotungstic acid and observed by transmission electron microscopy (*C*). Cells, lysate of cells; Mock, cells as negative control without transfection and infection; super, purified supernatants of culture medium of cells.

To evaluate the influence of ATP6V1A on RABV VLP production, we produced and detected rabies VLPs by transfecting plasmids expressing viral M and G protein, respectively (40). Western blotting analysis demonstrated that rabies VLPs consists of M and G proteins (Fig. 10*A*) and there is no apparent difference of M or G protein level between the VLPs produced in HEK293T cells depleted ATP6V1A or not (Fig. 10*A*). To detect the integrity of rabies VLPs, purified pellets were detected by Western blotting in non-denaturing and non-reducing conditions. Rabies VLPs were detected in the band larger than 250 kDa size, and depletion of ATP6V1A in cells has no obvious effect on the production of rabies VLPs (Fig. 10*B*). Then, we observed the morphology and specification of rabies VLPs by immunoelectron microscopy. Consistently with previous research (40), enveloped rabies VLPs particles with a diameter of approximately 200 nm were clearly visible, and several gold particles located on the surface of rabies VLPs when murine serum against viral G protein was used as the primary antibody (Fig. 10*C*). Knockdown of ATP6V1A in cells did not influence the morphology of rabies VLPs (Fig. 10*C*). These results showed that ATP6V1A has no effect on the production and morphology of rabies VLPs.

### Vero cells stably overexpressing ATP6V1A promote RABV replication

**Figure 11:**
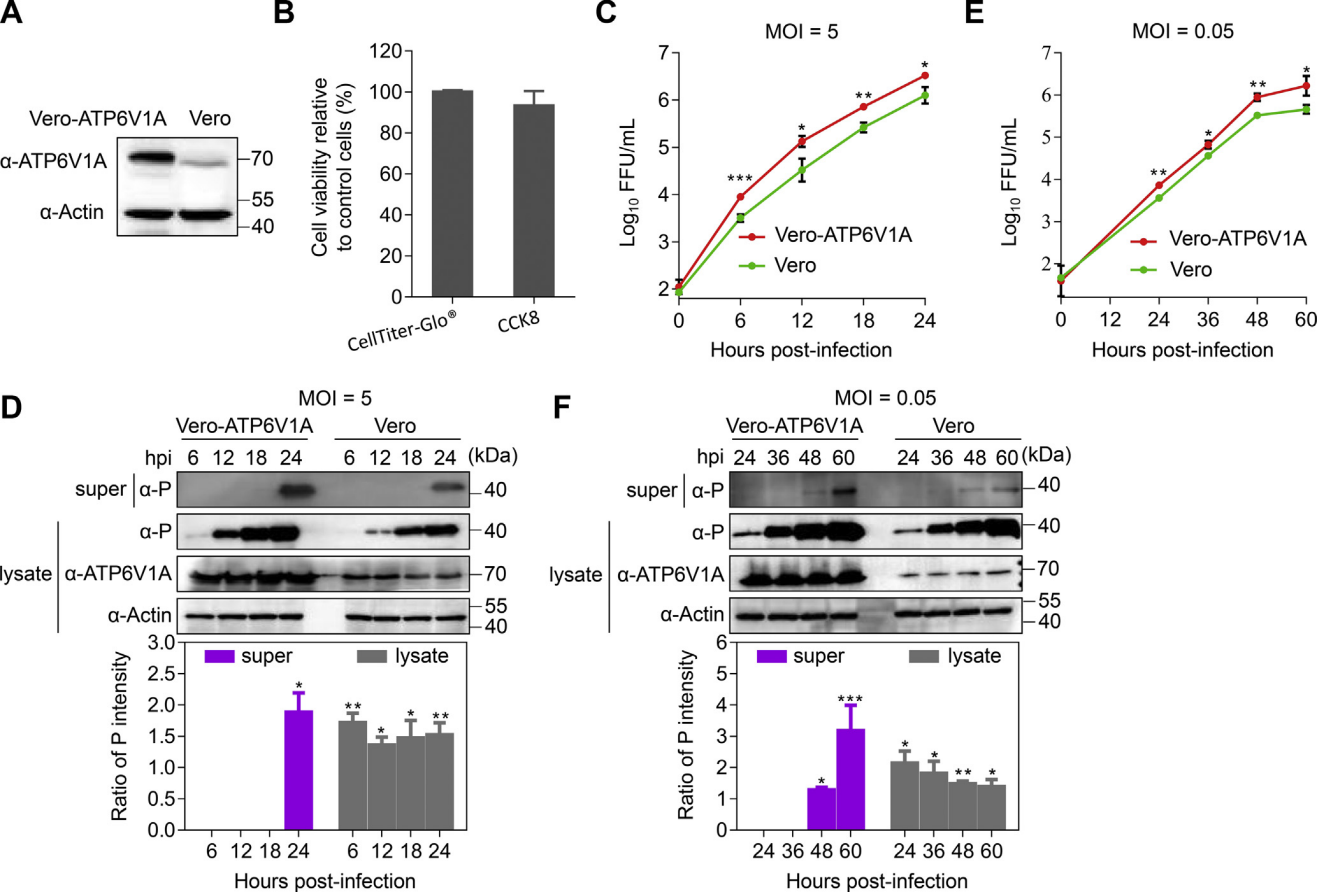
**Vero cell line stably overexpressing ATP6V1A promotes RABV replication.** Western blotting confirmed the overexpression of ATP6V1A in the stably transfected cell line Vero-ATP6V1A (*A*). Viability of Vero-ATP6V1A or Vero cells was determined by the quantification of ATP or NADH using CellTiter-Glo (Promega, USA) reagent or Cell Counting Kit-8 (CCK8) (Dojindo, China), respectively, at 48 h post seeding in 96-well plate. The relative assessments of cell viability were normalized by comparison with Vero cell as 100% cell viability (*B*). The culture medium of cells was collected and centrifuged (5000*g*/min, 10 min) at 4 °C at indicated time points post infection. Viral titers in the culture supernatants of Vero-ATP6V1A cells or Vero cells infected with ERA virus at an MOI of 5 (*C*) and 0.05 (*E*). ATP6V1A in the cell lysates and viral P protein in the culture supernatants or lysates of Vero-ATP6V1A cells or Vero cells infected with ERA virus at an MOI of 5 (*D*) or 0.05 (*F*) were detected by Western blotting. Statistical analysis of the P protein levels in the upper panel was performed by using ImageJ software. The value for the P protein was standardized to the β-actin level and normalized to that in Vero cells. Data were expressed as means ± SD of triplicate well from one independent experiment. Statistical significance was determined by ANOVA with a multiple comparison correction. ∗*p* < 0.05, ∗∗*p* < 0.01, ∗∗∗*p* < 0.001. Super, supernatants of culture medium of cells; lysate, lysate of cells.

The Vero (African green monkey kidney) cell line is the most widely accepted continuous cell line for vaccine production. It was introduced to produce the inactivated rabies vaccine in 1982 and has been in use for over 30 years (41). To examine whether the effect of ATP6V1A overexpression on RABV growth could be used to improve rabies vaccine production, we established a stable Vero cell line overexpressing ATP6V1A, Vero-ATP6V1A. ATP6V1A expression was substantially increased in Vero-ATP6V1A cells (Fig. 11*A*), and this overexpression did not affect cell viability determined by the quantification of ATP or NADH using CellTiter-Glo (Promega, USA) reagent or Cell Counting Kit-8 (CCK8) (Dojindo, China), respectively (Fig. 11*B*). We infected Vero-ATP6V1A and Vero cells with ERA virus at an MOI of 5 and found that viral titers in the culture supernatants of the Vero-ATP6V1A cells were 2.8- to 4.0-fold higher than those of Vero cells at different time points post infection (Fig. 11*C*). The level of P protein in the Vero-ATP6V1A cell supernatants was 1.9-fold higher than that in Vero cell supernatants at 24 h post infection (Fig. 11*D*). P protein in the lysates of Vero-ATP6V1A cells and Vero cells was detectable at 6 h post infection, and the P protein level was higher in the Vero-ATP6V1A cell lysates than in the Vero cell lysates at 24 h post infection (Fig. 11*D*). Steady overexpression of ATP6V1A in Vero-ATP6V1A cells infected with ERA virus at an MOI of 0.05 yielded about 4-fold increase in viral growth titers (Fig. 11*E*) and the P protein expression (Fig. 11*F*). These data suggest that overexpression of ATP6V1A in Vero cells promotes the propagation of RABV, which could be a potential strategy to improve the production of RABV vaccine.

## Discussion

The functions of RABV M protein in regulating the balance between viral transcription and translation (7) and virion assembly and budding (8) have been mostly characterized. The role of this protein in the early stage of infection, however, is not well known. Based on a clue from the protein interactome with RABV M protein, we found that host factor ATP6V1A interacts with the M protein and is important for RABV infection by affecting the dissociation of incoming viral M proteins during viral uncoating. The lysine residue at position 256 and the glutamic acid residue at position 279 of ATP6V1A determine its interaction with the M protein. We also demonstrated that ATP6V1A's promotion of RABV replication depends on its interaction with the M protein.

Dissociation of viral M proteins is an indication of enveloped virus uncoating, and the cellular distribution of dissociated M protein related with its function on viral early stage of life cycle (33). M proteins self-associate in the virion to maintain the virion's integrity; however, the M proteins in virions must dissociate during the uncoating step in order to release the viral genome into the host during viral infection (5, 42). Previous researches on vesicular stomatitis virus (VSV, another prototype of Rhabdoviridae family) showed that, when VSV M proteins separated from nucleocapsids, they were dissociated and diffusely distributed in cells during viral uncoating (43). M proteins may tightly associate with viral nucleocapsids before viral uncoating. The detergent (octylglucoside) at a low ionic strength removed the viral membrane containing G proteins but not M proteins, which tightly associated with nucleocapsids (44). Acid conditions did not enhance the separation of the VSV M proteins from nucleocapsids (45). Here we found that a small but detectable fraction of aggregated M proteins of RABV remained in the plasma membrane, and most of M puncta distributed in cytoplasm of the cells depleting ATP6V1A in acidic condition, which suggests that RABV uncoating does not occur in the absence of ATP6V1A, and thus M proteins still associate with nucleocapsids and do not diffuse in the cytoplasm. Further studies may be needed to explore the molecular mechanism of dissociation of RABV M protein and its separation from nucleocapsids during viral uncoating.

The uncoating of RABV occurs after viral particle entry into the host cell endosomes via endocytosis and membrane fusion (17, 46). A previous study indicated that low pH in the endosome is required for membrane fusion stimulated by conformationally changed RABV G protein (18). Given that ATP6V1A is a catalytic subunit of V-ATPase and the major player for acidification of endosome where RABV first enters, it seemed reasonable to think that knockdown of ATP6V1A would influence the membrane fusion of RABV. But our results showed that the interaction of the M protein with ATP6V1A was indispensable for RABV uncoating after membrane fusion, indicating that the uncoating of RABV is not a simply passive result after membrane fusion. It seems that the released M-bound nucleocapsids actively hijack the cytoplasmic V1 domain to promote its uncoating through the interaction of the M protein with ATP6V1A. It would also be of interest to discover whether other viruses that enter cells through receptor-mediated CME, including VSV (47) and influenza virus (48), use a similar molecular mechanism for uncoating.

The interaction between ATP6V1A and the M protein has functional relevance to RABV replication. The middle domain of ATP6V1A is critical for its interaction with the M protein. *Trans*-complementation with the interacting domain of ATP6V1A restores RABV infection as ATP6V1A did in knocked-down cells. Mutation of the lysine residue at position 256 and the glutamic acid residue at position 279 in the interacting domain of ATP6V1A destroyed the interaction activity with the M protein, which then blocked the ATP6V1A-induced promotion of RABV replication. It is interesting to note that the N-terminal domain of the M protein, which is responsible for its interaction with ATP6V1A, also contains the late budding domain PPEY motif that is responsible for efficient virion release and the pathogenicity of RABV (49), as well as amino acids 77 to 202, which are responsible for binding to the G protein (50). These findings highlight the importance of the N-terminal domain of the M protein in its multiple functional roles in RABV replication.

Promotion of viral replication by modulating the expression of host factors in cells may benefit vaccine manufacturing. Previous studies have shown that the host protein CD81 is required for pseudotyping retrovirus-like particle vaccine candidates with hepatitis C virus envelope protein E2, demonstrating the importance of the expression of host proteins in cell lines for the development of vaccines (51, 52). At present, four types of rabies vaccines are recommended by the WHO for human use: purified Vero cell vaccine (PVCV), purified chick embryo cell vaccine (PCECV), purified duck embryo vaccine (PDEV), and human diploid cell vaccine (HDCV) (53). Vero cells have been used to produce the inactivated rabies vaccine since 1982 (41, 53). In this study, we established Vero-ATP6V1A cells stably overexpressing ATP6V1A and found that the RABV particles yielded in the supernatant of Vero-ATP6V1A cells is about 4-fold than that yielded in the supernatant of Vero cells, which can be applied to decrease the cost of RABV vaccine production and make RABV vaccine more available for the people in developing areas such as Africa (54).

In summary, we found that ATP6V1A facilitates RABV replication by interacting with the viral M protein and affecting viral uncoating. Our results demonstrate that RABV M protein plays multiple roles in the viral life cycle including during the early stage of uncoating by interacting with host factors.

## Experimental procedures

### Cells and viruses

Human embryonic kidney cells (HEK293T, CRL-3216; ATCC or HEK293, CRL-1573; ATCC), baby hamster kidney cells (C-13; ATCC) stably expressing T7 polymerase (BSR-T7/5), Vero (African green monkey kidney) cells (CCL-81; ATCC), and a Vero cell line stably overexpressing ATP6V1A protein (Vero-ATP6V1A) were cultured in Dulbecco's modified Eagle's medium (Life Technologies, USA) with 10% fetal bovine serum (Life Technologies, USA).

RABV Evelyn-Rokitnicki-Abelseth (ERA) strain was maintained in our laboratory. Recombinant ERA expressing eGFP (ERA-eGFP) was generated as described previously (16).

### Plasmids construction

The FLAG-tag sequence was cloned into the expression plasmid pCAGGS (55) at either the N or C terminus, and named pNF and pCF, respectively. The ORF of the M protein of ERA was cloned into the plasmid pCAGGS in-frame with a FLAG-tag sequence at either the N- or C-terminal; the resultant constructs were named pM-NF and pM-CF, respectively. The ORF of the M protein was fused with GST at the C terminus and cloned into pGEX-4T-1 (56), and the resultant plasmid pGEX-M-GST was used with *Escherichia coli* to express and purify the M protein for pull-down assays. Two pCAGGS constructs were generated to express two truncates of the M protein, amino acids 1 to 76 and 77 to 202, respectively, which were fused to the C terminus of eGFP tagged with Myc and named pM1-76 and pM77-202, respectively. pcDNA plasmids (57) were constructed to express the M or G protein of ERA and named as pcM and pcG, respectively.

The Myc-tag sequence was cloned into pCAGGS and named pMyc. The ORF of ATP6V1A (Genelopoeia, China) was cloned into pCAGGS in-frame with a Myc-tag sequence at the C terminus and named pA-Myc. Four truncated constructs based on pCAGGS were generated to express four truncates of ATP6V1A, specifically amino acids 1 to 159, 160 to 309, 310 to 464, and 465 to 617, each of which was tagged with Myc at the C terminus, and named pA1-159, pA160-309, pA310-464, and pA465-617, respectively. The construct based on pA160-309 with the two mutations of K256Q and E279Q was named pMu160-309. The pCAGGS construct expressing amino acids 160 to 309 tagged with HA at the C terminus was named as pA160-309-HA. The pGEX-4T-1 (56) construct that expressed the ORF of ATP6V1A fused with His at the C terminus was generated as pA-His to express and purify ATP6V1A in *E. coli* for pull-down assays.

### Antibodies

The mouse anti-FLAG (Sigma-Aldrich, USA), mouse anti-Myc (Sigma-Aldrich, USA), rabbit anti-ATP6V1A (Sigma-Aldrich, USA), mouse anti-β-actin (Proteintech, China), mouse anti-HA epitope tag (Proteintech, China), mouse anti-GST (Sigma-Aldrich, USA), mouse anti-His epitope tag (Proteintech, China), rabbit IgG (Solarbio, China) and the secondary antibodies (HRP-conjugated anti-mouse IgG antibody [Sigma-Aldrich, USA], HRP-conjugated anti-rabbit IgG antibody [Genescript, China], FITC-conjugated goat anti-mouse IgG antibody [ZSGB. Bio, China], Alexa Fluor 647-labeled goat anti-rabbit IgG (H + L) [Beyotime, China]) and gold-labeled goat anti-mouse IgG antibody (Sigma-Aldrich, USA) were purchased from the indicated vendors. Murine sera against M, P, or G protein were produced in our laboratory.

### Co-IP, pull-down assay, and mass spectrometry

HEK293T cells were transfected with pM-NF or pM-CF, or the control plasmids pNF or pCF by using the TransIT-293 (Mirus, USA) transfection reagent according to the manufacturer's instructions. The cells were incubated with lysis buffer (50 mM Tris·HCl [pH 7.5], 150 mM NaCl, 1 mM EDTA, 0.5% Nonidet P-40, and protease inhibitor mixture Complete Mini [Roche, USA]) for 1 h at 4 °C at 24 h post transfection. Supernatants, clarified by centrifugation, were incubated with an anti-FLAG affinity gel (Sigma-Aldrich, USA) for 18 h at 4 °C. Proteins bound to the affinity gel were eluted with elution buffer (50 mM Tris·HCl [pH 7.5], 150 mM NaCl, and 0.5 mg/ml FLAG peptide [Sigma-Aldrich, USA]) for 1 h at 4 °C. Then, a 10% (vol/vol) volume of the eluted proteins was subjected to SDS-PAGE and silver staining (Thermo Invitrogen, USA); the remaining 90% (vol/vol) volume of eluted proteins was subjected to liquid chromatography tandem mass spectrometry (LC-MS/MS) analysis to identify coimmunoprecipitated host proteins. Samples were mixed with trypsin and digested at 37 °C overnight. LC-MS/MS was implemented by using an EASY-nLCTM 1200 UHPLC system (Thermo Invitrogen, USA) coupled with an Orbitrap Q Exactive HF-X mass spectrometer (Thermo Invitrogen, USA) at Shanghai Applied Protein Technology Co Ltd in China. The MS/MS signals were then processed against the Uniprot human protein database (155,558 sequences as of October 15, 2016) using the Mascot algorithm (version 2.2; Matrix Science) with the following parameters: dynamical modifications: oxidation (M); fixed modifications: carbamidomethyl: (C); maximum missed cleavages, 2; filter by score ≥20.

For the ERA-M and ATP6V1A coimmunoprecipitation (Co-IP) assay, HEK293T cells were cotransfected with plasmids expressing ERA-M protein or the M truncates, and plasmids expressing ATP6V1A protein or the ATP6V1A truncates by using TransIT-293 transfection reagent. At 24 h post transfection, the cells were lysed with ice-cold lysis buffer on a flip shaker for 1 h at 4 °C. Cell lysates were centrifuged (8000*g*/min, 10 min) at 4 °C, and clarified lysates were incubated with beads conjugated with anti-FLAG (Sigma-Aldrich, USA) or anti-Myc (Sigma-Aldrich, USA) affinity gel on a flip shaker for 18 h at 4 °C. Incubated beads were washed five times with lysis buffer, followed by SDS-PAGE and Western blotting.

For the ERA-M and ATP6V1A pull-down assays, ERA-M protein fused with C-terminal GST was expressed in *E. coli* and purified by using glutathione Sepharose 4B beads (GE, USA). ATP6V1A fused with a His tag at the C terminus was expressed in *E. coli* and purified by using a Ni-NTA column (GE, USA). For the GST pull-down assay, purified M-GST protein (10 μg) or GST protein at equimolar ratios was incubated with glutathione 4B-Sepharose beads on a flip shaker for 4 h at 4 °C. After three washes with lysis buffer, the beads were incubated with ATP6V1A-His protein (10 μg) on a flip shaker for 8 h at 4 °C. After three more washes with lysis buffer, the beads were assessed by Western blotting. For the His-tag pull-down assay, purified ATP6V1A-His protein (10 μg) was incubated with beads conjugated with His antibody on a flip shaker for 4 h at 4 °C. The beads were washed three times and incubated with M–GST proteins (10 μg) on a flip shaker for 8 h at 4 °C. The beads were then washed again and assessed by Western blotting.

### SiRNA knockdown of ATP6V1A

HEK293T cells were transfected with small interfering RNA (siRNA) pool oligonucleotides (SMARTpool from Dharmacon, USA) that targeted four different sequence domains of ATP6V1A (ATP6V1A siRNA) or were non-targeting to any gene in the human genome (scrambled siRNA) at a concentration of 30 nM by using the Lipofectamine RNAiMAX transfection reagent (Invitrogen, USA) according to the manufacturer's instructions. At 48 h post transfection, the cells were infected with ERA at the indicated MOI, and culture medium or lysates of cells were collected and centrifuged (5000*g*/min, 10 min) at 4 °C at the indicated time points post infection. Viral titers in the centrifuged culture medium were detected as focus forming unit per milliliter (FFU/ml) in BSR-T7/5 cells. The levels of viral P protein in the centrifuged culture medium, and P protein, ATP6V1A, or β-actin in the lysate of cells were detected by Western blotting.

### Detection of incoming RABV M proteins

HEK293T cells were transfected with ATP6V1A siRNA or scrambled siRNA or were treated with 0.1 μM bafilomycin A1 (BAF A1; Millipore, Australia). At 48 h post transfection, the cells were incubated with 5 μM protein synthesis inhibitor cycloheximide (CHX; APExBIO, USA) for 1 h at 37 °C and then incubated with ERA virus at an MOI of 100 for 1 h at 37 °C in the presence of CHX. Unabsorbed virus was removed by replacing the medium three times with fresh medium, and the cells were incubated for an additional 2 h at 37 °C. Note that CHX and/or BAF A1 were maintained on the cells throughout the incubation. Cells were fixed and permeabilized and then incubated with mouse anti-M protein serum and rabbit anti-ATP6V1A antibody (Sigma-Aldrich, USA). After staining with FITC-labeled anti-mouse secondary antibody (ZSGB.Bio, China) and Alexa Fluor 647-labeled goat anti-rabbit IgG (H + L) (Beyotime, China) for 1 h, the cells were examined by means of confocal microscopy with an LSM880 system (Carl Zeiss, Germany).

### Acid bypass assay

HEK293T cells were transfected with ATP6V1A siRNA or scrambled siRNA or treated with 0.1 M BAF A1 for 1 h before infection with ERA. At 48 h post transfection, the cells were incubated with 5 μM CHX for 1 h at 37 °C and then infected with ERA virus at an MOI of 100. The cells were treated with neutral PBS or acidic PBS as previously described, respectively (38). For *trans*-complementation assay, at 48 h post transfection of siRNA, the cells were transfected with plasmids of pA-Myc or pA160-309 or pMu160-309 or pMyc, respectively. At 24 h post transfection, the cells were infected with ERA virus at an MOI of 100 and treated as previously described (38).

### Production and detection of rabies VLPs

A total of 9 ˣ 10^6^ HEK293T cells in a 10-cm dish were transfected with 15 μg of pcM and pcG at a molar ratio of 6:1 by using the TransIT-293 (Mirus, USA) transfection reagent according to the manufacturer's instructions and cultured for 48 h. Culture supernatants were harvested and ultracentrifuged as previously described (58). Purified rabies VLPs were separated by 4% to 12% SDS-PAGE under denaturing or non-denaturing and non-reducing conditions and analyzed by Western blotting with specific antibodies against M or G protein, respectively. The morphology and specification of rabies VLPs was observed by immunoelectron microscopy with specific mouse anti-G antibody as described previously (40).

### Establishment of a stable Vero cell line overexpressing ATP6V1A

The ORF of ATP6V1A was cloned into pQCXIN (Addgene, USA) to generate packaged retroviruses in HEK293 cells following the manufacturer's instructions and by using TransIT-Lenti Transfection Reagent (Mirus bio, USA). Vero cells were transduced with packaged retroviruses and cultured in medium supplemented with puromycin for selection. The surviving cell clone was isolated, propagated, and examined for stable expression of ATP6V1A by Western blotting.

### Cell viability assay

For siRNA interfered cells' viability measurement, HEK293T cells were seeded in opaque-walled 96-well plates and transfected with siRNAs. At 48 h post transfection, the viability of cells in culture were determined based on the quantification of ATP by using CellTiter-Glo (Promega, USA) reagent. A volume of 100 μl of CellTiter-Glo reagent was added directly into each well by following the manufacturer's instruction, and the contents were mixed for 10 min on a shaker at room temperature to induce cell lysis. After stabilizing luminescence signal at room temperature for 10 min, luminescence was measured with a GloMax 96 microplate luminometer (Promega, USA). For the viability measurement of Vero cells stably overexpressing ATP6V1A (Vero-ATP6V1A), Vero-ATP6V1A cells and Vero cells were seeded in opaque-walled 96-well plates. Twenty-four hours later, cells were treated with CellTiter-Glo reagent and luminescence was measured according to the manufacture's instruction.

Cell viability was also determined based on the quantification of NADH by using Cell Counting Kit-8 (CCK8) (Dojindo, China) according to the manufacturer's instruction. Cells were seeded in 96-well plates and transfected with siRNAs. At 48 h post transfection, culture medium was removed, 10 μl CCK8 solution mixed with 90 μl fresh medium was added and incubated for 2 h in 37 °C. The absorbance was measured at 450 nm. For the viability measurement of Vero cells stably overexpressing ATP6V1A (Vero-ATP6V1A), Vero-ATP6V1A cells and Vero cells were seeded onto 96-well plates. Twenty-four hours later, cells were treated with 10 μl CCK8 solution mixed with 90 μl fresh medium and absorbance was measured at 450 nm.

### Statistics

All experiments were performed two or three times, statistical analysis was performed for each experiment every time, and the data from one independent experiment were shown. Results were analyzed for statistical significance by using Student's *t* test. A *p* value of 0.05 was considered statistically significant.

## Data availability

The mass spectrometry proteomics data have been deposited to the ProteomeXchange Consortium via the PRIDE (59) partner repository with the location (/nfs/pride/drop/pride-drop-001/liuxingshenglin_20200821_032146) and the dataset identifier PXD021039. The arranged mass spectrometry proteomics data were deposited in the IMEx consortium through IntAct with the accession code IM-27674 (http://www.imexconsortium.org). All other data are contained within the manuscript and also deposited to Zenodo with accession number 10.5281/zenodo.4279152.

## Conflict of interest

The authors declare that they have no conflicts of interest with the contents of this article.The corresponding author had full access to all the data and accepts the final responsibility for the decision to submit this manuscript for publication.
